# Differential effects of physical activity on behavioral and prefrontal responses during repetitive inhibitory control in older adults

**DOI:** 10.3389/fnagi.2025.1684331

**Published:** 2025-10-15

**Authors:** Jae-Hoon Lee, Minchul Lee, Min-Seong Ha

**Affiliations:** 1 Department of Sport Science, College of Arts and Sports University of Seoul, Seoul, Republic of Korea; 2 Department of Sports Medicine, College of Health Science CHA University, Pocheon-si, Republic of Korea; 3 Laboratory of Sports Conditioning: Nutrition Biochemistry and Neuroscience, Department of Sport Science, College of Arts and Sports University of Seoul, Seoul, Republic of Korea

**Keywords:** physical activity, prefrontal cortex, fNIRS, inhibitory control, older adults

## Abstract

**Objectives:**

This study aimed to investigate the effects of physical activity levels on response time and prefrontal activation during repetitive performance of the Stroop task in older adults.

**Methods:**

We examined how physical activity influences selective inhibition and neurophysiological responses in the prefrontal cortex. Community-dwelling adults aged 65–85 years were classified into physically active (PA, *n* = 18) and physically inactive (PI, *n* = 19) groups using the International Physical Activity Questionnaire (IPAQ). On a single experimental day, participants completed the Stroop task in three consecutive blocks within one session. Participants completed the Stroop task three times. Reaction time and oxygenated hemoglobin (HbO) levels in the prefrontal cortex were measured using functional near-infrared spectroscopy (fNIRS) and analyzed by the Brodmann area.

**Results:**

A two-way repeated-measures analysis of variance revealed significant time, group, and interaction effects on reaction time (*p* < 0.05). The PA group showed a significant decrease in reaction time across repetitions (*p* < 0.001), whereas no such change was observed in the PI group. In terms of brain activation, HbO levels increased significantly over time in the left dorsolateral prefrontal cortex and the right ventrolateral prefrontal cortex (*p* < 0.05), although no significant group differences were observed.

**Conclusion:**

Regular physical activity may enhance cognitive adaptability and selective inhibition in older adults. Behavioral improvements were more evident than neural differences between the groups, highlighting the potential of everyday physical activity to support cognitive resilience in aging. This study provides neurophysiological evidence supporting the integration of physical activity into cognitive intervention strategies for older populations.

## Introduction

Rising global life expectancy has accelerated the transition to an aging society, making age-related cognitive decline an urgent public health concern (Brito et al., 2023). Cognitive aging is characterized by gradual deterioration in fundamental domains, such as attention, memory, and perceptual processing, which collectively lead to deficits in executive function, which is often considered the most vulnerable cognitive domain in older adults (Glisky, 2007). Executive dysfunction is a common cognitive deficit observed in dementia and may serve as an early marker of its progression (Alvarez and Emory, 2006; Royall et al., 2004; Tabert et al., 2006).

At the neurophysiological level, aging is accompanied by increased inflammation and oxidative stress, both of which impair neurovascular coupling (NVC), which is defined as the dynamic mechanism through which neuronal activation elicits hemodynamic responses, resulting in localized increases in cerebral blood flow that match the metabolic and energetic demands of active neural tissue (Iadecola, 2017). Evidence from human and translational studies indicates that aging is associated with impaired NVC and related cerebrovascular dysfunction, in part via oxidative stress and low-grade inflammation (Csipo et al., 2019). Disruption of NVC may contribute to diminished cognitive performance, particularly in executive tasks that rely heavily on the prefrontal cortex. Physical activity may mitigate these effects by enhancing NVC function and supporting cerebral perfusion (He et al., 2023).

Extensive meta-analyses and empirical studies supported the role of physical activity in preserving and enhancing cognitive performance in aging populations (Northey et al., 2018; Erickson et al., 2015). Acute bouts of aerobic exercise improve inhibitory control, although the underlying neural mechanisms may differ with age. For instance, younger adults tend to exhibit increased arousal following exercise, whereas older adults show enhanced activation of the mid-prefrontal cortex (Fujihara et al., 2021). Chronic interventions are similarly effective; 3 months of low-intensity aerobic training significantly improved reaction times during performance of the Stroop task and improved neural efficiency in older adults (Byun et al., 2024). In a seminal meta-analysis, Colcombe and Kramer (2003) highlighted that aerobic exercise yields the greatest benefits for executive function and that frequency, intensity, and time duration modulate its efficacy. They proposed that physical activity enhances neuroplasticity and serves as a critical buffer against cognitive aging.

However, the majority of the current literature is based on structured exercise interventions conducted in controlled environments. How habitual, real-world physical activity levels influence executive performance and the associated neural responses in cognitively healthy older adults remains unclear.

The Stroop task, which is widely used in cognitive aging research, is a robust measure of core executive functions, such as attentional control, inhibitory processing, and cognitive flexibility (MacLeod, 1991). Older adults typically exhibit exaggerated interference effects, making this task particularly relevant for detecting age-related changes (Idowu et al., 2024). Functional near-infrared spectroscopy (fNIRS) studies demonstrated that Stroop performance increases oxygenated hemoglobin (HbO) concentrations in the prefrontal cortex. Interestingly, after an exercise intervention, the same task elicits lower prefrontal activation, indicating enhanced neural efficiency (Byun et al., 2024). Additionally, neuroimaging studies suggested that Stroop performance involves a cross-hemispheric circuit between the left prefrontal cortex and the right cerebellum, enabling goal-directed behavior under conditions of high cognitive interference (Okayasu et al., 2023).

In cognitively intact older adults, repeated performance of the Stroop task results in improved behavioral performance and reduced prefrontal activation, reflecting practical effects and neural adaptation (Davidson et al., 2003). Recent studies in young adults suggested that, although physical activity may shorten reaction times during executive tasks, it does not necessarily alter prefrontal HbO levels (Tan et al., 2024). By contrast, systematic reviews have reported that prefrontal compensatory activation during executive tasks, such as the Stroop and Simon tasks, is evident in older adults, although such responses diminish with advancing cognitive decline (Cipriani et al., 2025). Prospective cohort studies further linked moderate-to-high levels of physical activity to a reduced incidence of cognitive impairment (Yaffe et al., 2001; Sofi et al., 2011). However, these studies primarily focused on individuals with mild cognitive impairment or high dementia risk. Experimental evidence on how everyday physical activity levels modulate executive functioning and neural efficiency in healthy, non-impaired, and older adults is lacking.

Therefore, this study aimed to investigate the influence of real-life physical activity levels on behavioral performance and prefrontal cortex activation during repeated Stroop task executions in cognitively normal middle-aged and older adults. Using fNIRS, we assessed whether higher physical activity levels enhance neural efficiency and facilitate executive adaptability, as evidenced by the practice effects on repeated task performance. By bridging behavioral and neurophysiological data, this study critically elucidates the preventive role of physical activity in cognitive aging and offers evidence to support individualized activity-based interventions for maintaining executive function in older adults.

## Materials and methods

### Participants

This study recruited community-dwelling older adults aged 65–85 years using public advertisements. Participants who voluntarily expressed their willingness to participate were included only after being fully informed of the study procedures and signing informed consent forms. This analysis was part of a secondary study utilizing existing data originally collected to examine the effects of a physical activity intervention. The current investigation focused on analyzing practice effects and prefrontal activation patterns during repetitive Stroop tasks based on physical activity levels.

The sample size was estimated using G*Power 3.1 (Heinrich Heine University Düsseldorf, Düsseldorf, Germany) for a repeated-measures analysis of variance (Luna et al., 2008). Assuming a medium effect size of *f* = 0.25, a significance level of *α* = 0.05, and a power of 1–*β* = 0.90, a minimum of 36 participants was required. Initially, 40 participants were recruited to account for potential attrition. Two participants withdrew from the study, and 38 participants were included in the final analysis (Table 1).

**Table 1:** Characteristics of participants.

Variables	PA (m = 8, *f* = 10)		PI (m = 3, *f* = 16)	
	M	SD	M	SD
Age (years)	76.44	4.79	73.16	5.53
Height (m)	1.59	0.10	1.56	0.07
Weight (kg)	59.49	8.84	59.95	7.91
BMI (kg/m^2^)	23.67	2.61	24.74	2.88
Body Fat (%)	31.44	7.30	36.85	6.35
MoCA	23.06	4.05	21.16	3.86

Values are presented as means (M) ± standard deviations (SD). PI, physically inactive group; PA, physically active group; BMI, body mass index; MoCA, Montreal Cognitive Assessment.

The inclusion criteria were an absence of neurological disorders, psychiatric conditions, and chronic alcohol or drug misuse within the past 6 months. Sensory function was assessed using the Weber and Rinne tests for hearing (Kelly et al., 2018) and a Snellen chart for vision. Participants were required to have sufficient sensory capacity to engage in the task, and only those who met this criterion were included in the study. The study protocol was approved by the Institutional Review Board of Dongguk University (DUIRB-202208-10) and was conducted in accordance with the Declaration of Helsinki.

### Study design

This study used a cross-sectional design and classified the participants into two groups based on their physical activity levels. Activity status was determined using the International Physical Activity Questionnaire (IPAQ). Participants were categorized as physically active (PA) if they engaged in at least 150 min of moderate-intensity physical activity or 75 min of vigorous-intensity physical activity per week; otherwise, they were assigned to the physically inactive (PI) group.

All participants rested for 10 min prior to the experiment; this preparatory rest was excluded from the analysis. Subsequently, participants performed a color-word Stroop task across three consecutive blocks, each consisting of 18 trials (Table 2). A 3-min resting-state fNIRS recording immediately preceding the Stroop task was used as the physiological baseline for analysis. Between blocks, 15-s intervals were provided to minimize fatigue and allow hemodynamic recovery. During task performance, participants wore an fNIRS device (NIRSIT; OBELAB Inc., Seoul, Republic of Korea) to measure prefrontal cortical activation. Data were collected using a block design comprising resting and task periods. The mean oxyhemoglobin (oxy-Hb) levels were extracted from each block and referenced to the 3-min pre-task baseline for statistical analysis.

**Table 2:** Experimental procedures.

Phase	Task type	Timing/questions	Notes
Pre-task rest	Resting state	10 min	Not included in the analysis
Baseline	Eyes open test	3 min	Used as a physiological baseline
Stroop Block 1	Stroop task	18 trials × 3.5 s each	~15 s inter-block rest after completion
Stroop Block 2	Stroop task	18 trials × 3.5 s each	~15 s inter-block rest after completion
Stroop Block 3	Stroop task	18 trials × 3.5 s each	End of task

### Stroop task

To assess executive function, specifically inhibitory control and attentional regulation, a color-word Stroop task was administered. This task is a widely validated neuropsychological paradigm strongly associated with dorsolateral prefrontal cortex (DLPFC) activity (MacLeod, 1991).

Participants viewed Korean stimuli (“빨강” [red], “파랑” [blue], or neutral “XXXX”) on a computer screen and were instructed to respond based on the color of the text, not the meaning of the word. A left-button response was required for red, and a right-button response was required for blue. This incongruent condition created a demand for cognitive inhibition (Table 3). The task included three blocks of 18 trials (54 trials in total), with each stimulus being displayed for 3.5 s. All the responses were recorded using the Python-based software (Python Software Foundation, Wilmington, DE, United States). The task structure was as follows: number of blocks = 3, number of trials per block = 18, and stimulus duration = 3.5 s. The participants completed a practice block before the actual task, and both reaction time and accuracy were used for the analysis.

**Table 3:** Stimuli used in the Korean version of the Stroop task.

Type	Example	Description
Neutral	XXXX in red or blue	Non-verbal stimulus. The participant responds based only on the color
Congruent(RED)	“빨강” in red	The word’s meaning and font color are the same
Congruent(BLUE)	“파랑” in blue	The word’s meaning and font color are the same
Incongruent(RED)	“파랑” in red	The word’s meaning and font color are mismatched.
Incongruent(BLUE)	“빨강” in blue	The word’s meaning and font color are mismatched.

### Assessment of physical activity

Physical activity levels were quantified using the IPAQ, a validated self-report tool widely used in geriatric research for its reliability and sensitivity to habitual exercise behavior (Tomioka et al., 2011). Based on weekly activity, participants who met the guidelines (≥150-min moderate or ≥75-min vigorous activity) were classified as part of the physically active (PA) group, whereas those below the threshold were categorized as part of the physically inactive (PI) group. To confirm that the two groups were clearly distinct in their physical activity levels, we compared weekly minutes of moderate-to-vigorous physical activity (MVPA) between the groups. The PA group reported significantly greater MVPA (M = 252.22 min, SD = 127.63) than the PI group (M = 24.74 min, SD = 43.12), t (35) = − 7.667, *p* < 0.001.

### Cognitive screening

Global cognitive function was screened using the Korean version of the Montreal Cognitive Assessment (MoCA-K), which was adapted by Lee et al. (2008) from the original MoCA developed by Nasreddine et al. (2005). This tool evaluates multiple cognitive domains, including visuospatial/executive function, language, attention, memory, abstraction, delayed recall, and orientation. A cutoff score of 23 yields 70% sensitivity and 92% specificity for mild cognitive impairment (Park et al., 2010). An additional block was added for participants with ≤6 years of formal education to control educational biases. In this study, the MoCA-K was administered to characterize the cognitive profile of the sample; participants were not excluded based on cognitive performance.

### fNIRS acquisition and preprocessing

Cortical hemodynamic responses were acquired using the NIRSIT system (NIRSIT; OBELAB Inc., Seoul, Republic of Korea), which employs near-infrared light at wavelengths of 780 and 850 nm. The system consisted of 24 sources and 32 detectors, yielding 48 channels with a spatial resolution of approximately 3 cm and a sampling rate of 8.138 Hz (Choi et al., 2016). The probe was centered on the forehead, and recordings were conducted in a dimly lit room to reduce light interference. Signal quality was assessed prior to data acquisition. Raw signals were transformed into oxy-Hb concentrations using the modified Beer–Lambert law. A bandpass filter (0.005–0.1 Hz) was used to remove the physiological and environmental noise. Although the NIRSIT system uses 780 nm as the lower wavelength—slightly longer than the conventional 760–770 nm range commonly employed in other fNIRS devices—this setting remains within the near-infrared optical window and is technically acceptable. We acknowledge this characteristic as a methodological consideration for future research.

### fNIRS data analysis

The fNIRS data were preprocessed and analyzed using NIRSIT QUEST software (OBELAB Inc., Seoul, Republic of Korea). The fNIRS signals were segmented into task and rest epochs for each trial. Preprocessing steps included spike removal, baseline correction, and channel exclusion based on specific quality control criteria. Channels were excluded from the analysis if they exhibited a mean signal intensity below 30 (Yücel et al., 2021), a coefficient of variation greater than 15% (Pfeifer et al., 2018), more than 5% repetition of identical values across the block series, or a strong negative correlation (*r* < −0.9) between oxy-Hb and deoxyhemoglobin signals (Takizawa et al., 2014). In addition, motion artifacts were identified and either corrected or removed using the temporal derivative distribution repair algorithm (Fishburn et al., 2019).

Following preprocessing, a general linear model was applied to quantify task-evoked changes in oxy-Hb concentrations. These analyses were performed across individual channels and aggregated within the Brodmann areas. Statistical significance was set at a *p*-value of < 0.05.

**Figure 1:**
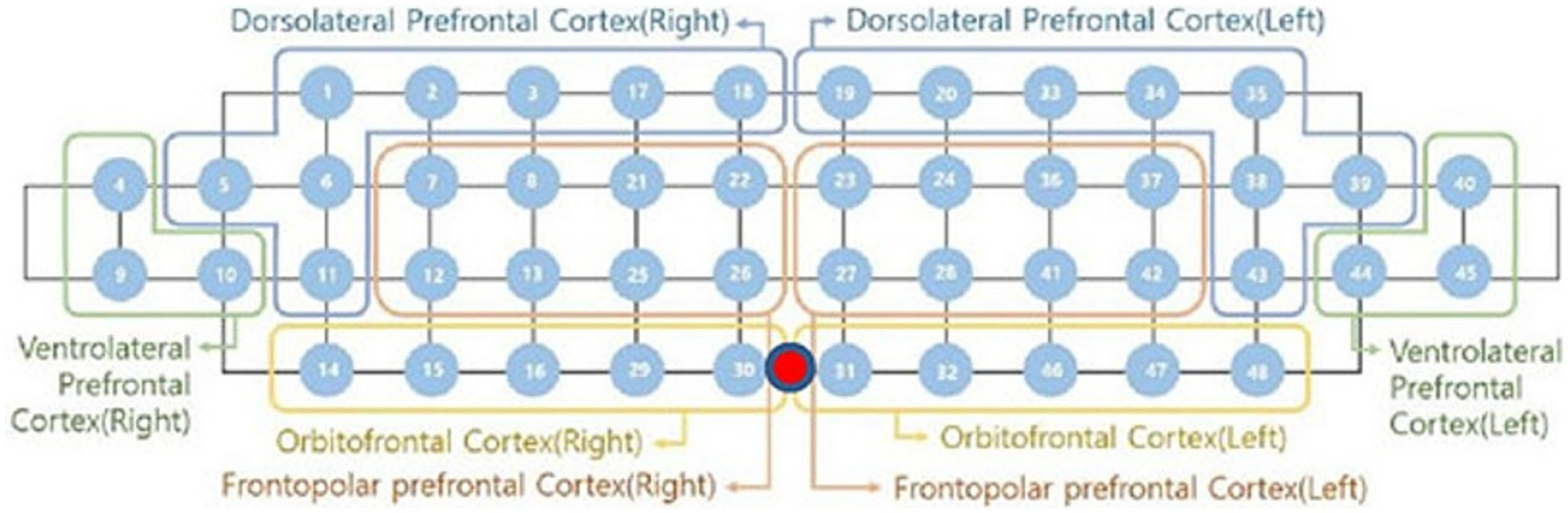
fNIRS channel configuration for prefrontal cortex subregions. From: athlete-specific neural strategies under pressure: a fNIRS pilot study (Park et al., 2020).

To visualize cortical activation, activation maps were generated based on oxy-Hb responses during the Stroop task (Figure 1). The 48 channels were anatomically grouped into eight regions corresponding to Brodmann areas: the DLPFC, the ventrolateral prefrontal cortex (VLPFC), the orbitofrontal cortex (OFC), and the frontopolar prefrontal cortex (FPC) for both the left and right hemispheres (Brodmann, 1909).

### Statistical analysis

Statistical analyses were performed using SPSS version 28.0 (IBM Corp., Armonk, NY, United States). Descriptive statistics are reported as means and standard deviations (M ± SD). Data normality was verified using the Shapiro–Wilk test. A one-way repeated-measures ANOVA was used to examine within-subject effects across Stroop blocks. To assess group (PA vs. PI) and block (Brito et al., 2023; Glisky, 2007; Alvarez and Emory, 2006) interactions, a 2 × 3 repeated-measures ANOVA was conducted. Bonferroni-adjusted *post-hoc* tests were used for significant effects. The significance level was set at a *p*-value of < 0.05 for all analyses.

## Results

### Stroop reaction time

**Figure 2:**
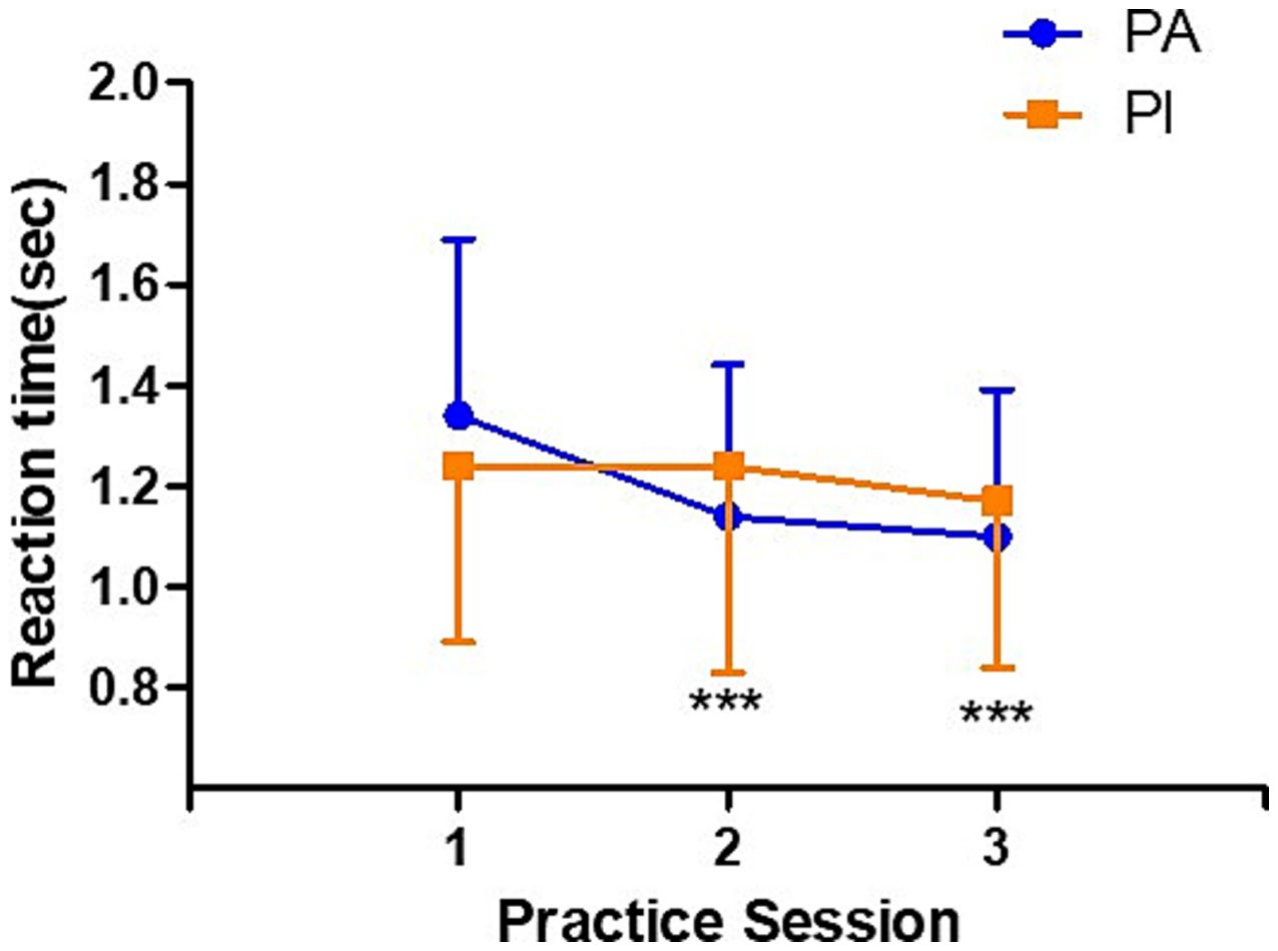
Stroop practice task reaction time. Changes in reaction time during the Stroop practice task across three blocks in the physically active group (PA, *n* = 18) and the physically inactive group (PI, *n* = 19). Data are presented as means ± standard deviations. For clarity, only the upward error bars are shown for the PA group, and only the downward error bars are shown for the PI group. The PA group showed significant reductions in reaction time at practices 2 and 3. Significant main effects of time (*F* = 8.212, *p* = 0.001), group (*F* = 4.676, *p* = 0.038), and a time × group interaction (*F* = 4.194, *p* = 0.019) were observed. Abbreviations: PA, physically active; PI, physically inactive. ***p < 0.001 vs. practice 1.

Figure 2 presents the results of the reaction time analysis for the color-word Stroop task, which was administered three times to participants classified into the PI and PA groups based on their physical activity levels. A repeated-measures analysis revealed significant main effects of time (*F*(2, 96) = 8.212, *p* = 0.001) and group (*F*(1, 48) = 4.676, *p* = 0.038), as well as a significant interaction between time and group (F(2, 96) = 4.194, *p* = 0.019).

*Post-hoc* analysis using Bonferroni correction indicated that, within the PA group, reaction times in both the second (*p* < 0.001) and third trials (*p* < 0.001) were significantly shorter than those in the first trial, suggesting improved cognitive performance with repeated exposure to the task. In contrast, the PI group showed no significant changes in reaction time across the three repetitions, indicating limited cognitive adaptation to the task in the absence of sufficient physical activity.

### Prefrontal cortex activation

**Figure 3:**
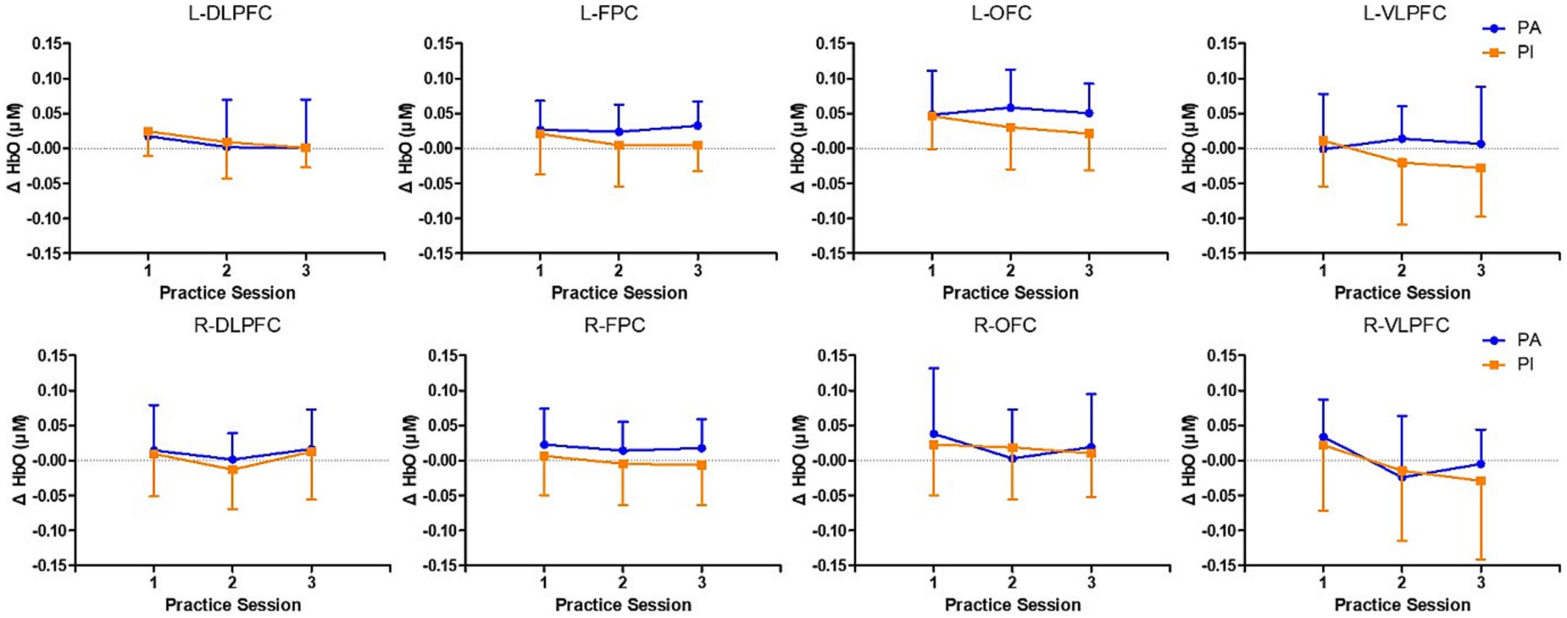
Changes in oxygenated hemoglobin levels in Brodmann areas across practice blocks in the physically active and inactive groups. Changes in oxygenated hemoglobin levels (μM) across Brodmann areas over three practice blocks in the physically active group (PA, *n* = 18) and the physically inactive group (PI, *n* = 19). The analyzed regions included the left and right dorsolateral prefrontal cortex (DLPFC), frontopolar cortex (FPC), the orbitofrontal cortex (OFC), and the ventrolateral prefrontal cortex (VLPFC). Data are presented as means ± standard deviations. For clarity, only the upward error bars are shown for the PA group, and only the downward error bars are shown for the PI group. A significant main effect of time was observed only in the left DLPFC (*F* = 3.493, *p* = 0.037) and the right VLPFC (*F* = 4.548, *p* = 0.015), indicating changes in oxygenated hemoglobin levels over time irrespective of group. No significant group effects or time × group interactions were observed in any region. Abbreviations: PA, physically active; PI, physically inactive; DLPFC, dorsolateral prefrontal cortex; FPC, frontopolar cortex; OFC, orbitofrontal cortex; VLPFC, ventrolateral prefrontal cortex.

**Figure 4:**
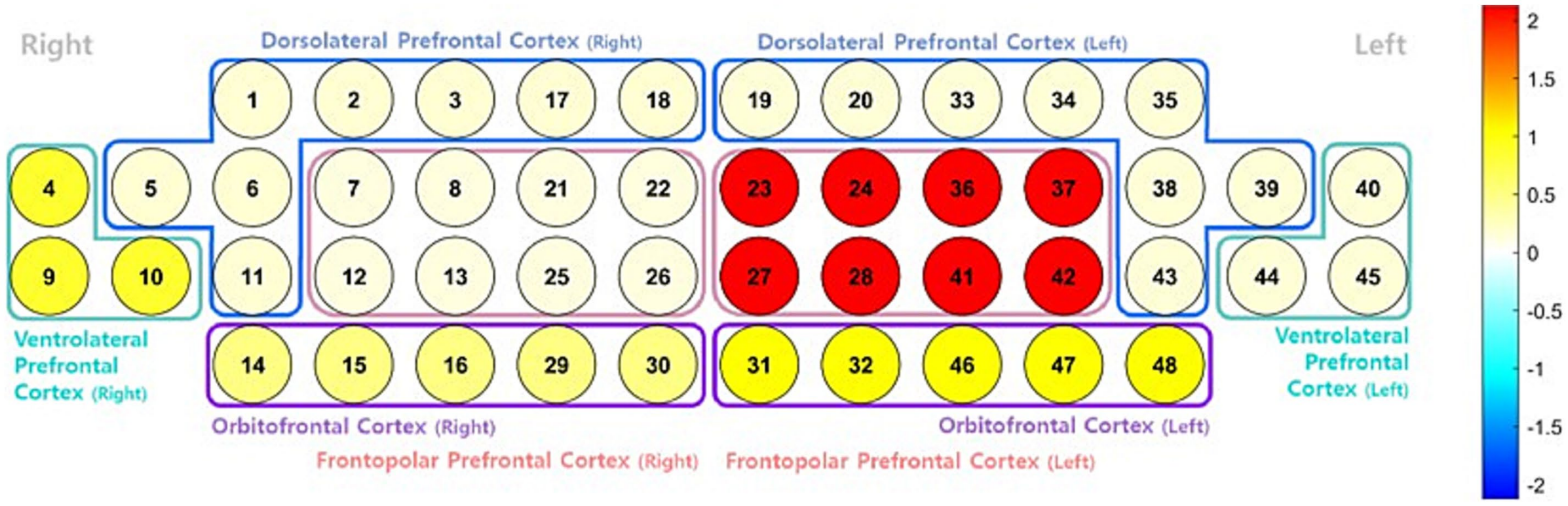
Surface rendering of the prefrontal cortex showing the interaction effect (group × condition) from a two-way analysis of variance (ANOVA) on oxyhemoglobin (HbO) concentration during repeated performance of the Stroop task. The color map represents the relative strength of the interaction effect across regions, with warmer colors (e.g., red) indicating higher contrast values and cooler colors (e.g., blue) indicating lower values. The color does not directly reflect statistical significance. Region-specific analyses revealed significant main effects of time in the left dorsolateral prefrontal cortex (DLPFC; F = 3.493, *p* = 0.037) and the right ventrolateral prefrontal cortex (VLPFC; F = 4.548, *p* = 0.015), with no significant group or interaction effects observed.

Figures 3, 4 illustrate changes in prefrontal cortical activation, as assessed via fNIRS, during repeated performance of the Stroop task. Activation was analyzed according to Brodmann areas to compare the PI and PA groups. A two-way repeated-measures ANOVA was conducted to assess the effects of time (three repetitions), group (PI vs. PA), and their interaction.

In the left DLPFC, a significant main effect of time was observed (*F*(2, 96) = 3.493, *p* = 0.037), indicating increased neural engagement with task repetition, regardless of group. However, neither the main effect of the group nor the time × group interaction was statistically significant in this region. Similarly, in the right VLPFC, a significant main effect of time emerged (F(2, 96) = 4.548, *p* = 0.015), again without significant group or interaction effects. Thus, task repetition alone may enhance activation in specific prefrontal regions, independent of the physical activity level.

No significant main effects or interactions were observed in other prefrontal areas, including the OFC and the FPC, suggesting that the observed activation changes across the prefrontal cortex were region-specific rather than global.

## Discussion

This study examined the effects of physical activity levels on behavioral performance and prefrontal cortical activation during repeated Stroop task execution in older adults. By comparing changes in response time and HbO levels using fNIRS, we aimed to elucidate how physical activity influences selective inhibition and neurophysiological responsiveness in the prefrontal cortex.

We observed significant main effects of time and group, along with a significant time × group interaction for reaction time. *Post-hoc* analysis indicated that participants in the PA group exhibited a significant reduction in response time across repeated task trials, whereas those in the PI group showed no such improvement. Thus, higher physical activity levels may enhance cognitive adaptability and learning during repetitive inhibitory control tasks in older adults.

Regarding prefrontal activation, significant main effects of time were observed in the left DLPFC and right VLPFC, although no significant group differences were observed between the groups or in their interactions. Although HbO concentrations showed a trend of increasing activation over time, specific pairwise comparisons between trials did not reach statistical significance. This pattern may reflect gradual neurophysiological adaptation. However, the magnitude and direction of the changes were insufficiently consistent to confirm robust group-level neural differences.

Improvements in response time likely reflect a combination of practice effects and enhanced cognitive flexibility (Davidson et al., 2003). The more pronounced behavioral gains observed in the PA group may indicate increased sensitivity to attentional regulation and inhibitory control, which aligns with previous research linking regular physical activity to improvements in executive functioning, particularly in tasks requiring cognitive shifting and flexible attentional engagement (Fujihara et al., 2021).

Emerging evidence emphasizes the qualitative dimension of physical activity. Physical activity can be classified as open-skilled (requiring dynamic adaptation to unpredictable environments) or closed-skilled (performed in stable and predictable contexts). Open-skilled activities offer superior benefits for inhibitory control, cognitive flexibility, and visuomotor tracking compared to closed-skilled or sedentary behaviors (Ingold et al., 2020). For instance, a 16-week quasi-experimental study of young adults has demonstrated that participation in open-skilled sports, such as badminton and basketball, yields greater improvements in executive functions, particularly inhibition and cognitive shifting, than closed-skilled activities (Li et al., 2024). These differences may stem from the varied cognitive demands and neural resources recruited by different types of exercise, suggesting that dynamic, complex physical activity may serve as an effective cognitive stimulus for tasks involving higher-order executive control tasks, such as Stroop tasks.

Systematic reviews further support the notion that physical activity, regardless of cognitive status, contributes positively to cognitive maintenance in older adults. Aerobic exercise in particular enhances prefrontal-based executive function and improves neural efficiency, suggesting that even routine physical activity in daily life may foster cognitive resilience (van Uffelen et al., 2008).

The absence of significant group differences in prefrontal activation can be attributed to several factors. First, fNIRS is limited to detecting hemodynamic changes on the cortical surface and cannot measure activity in subcortical structures, such as the hippocampus or anterior cingulate cortex (Leff et al., 2011). Second, despite similar behavioral outcomes, individuals may engage in distinct neural strategies, possibly reflecting compensatory mechanisms or individualized patterns of cognitive resource recruitment (Park and Reuter-Lorenz, 2009). Therefore, the lack of observed neural differences does not preclude the presence of underlying cognitive benefits. Instead, it may suggest differential neural efficiency or the employment of compensatory activation patterns.

Aging brains often exhibit structural and functional decline, yet maintain performance through the compensatory engagement of alternative neural circuits. These phenomena are encapsulated in the Compensation-Related Utilization of Neural Circuits Hypothesis and the Hemispheric Asymmetry Reduction in Older Adults Hypothesis (Reuter-Lorenz and Cappell, 2008; Cabeza, 2002). In the current study, the PI group maintained comparable levels of prefrontal activation despite no significant behavioral improvement, potentially reflecting a compensatory mechanism to preserve task performance. This finding aligns with the notion that aging brains recruit additional resources to sustain their cognitive output in response to functional limitations.

This study provides experimental evidence that higher levels of physical activity positively influence cognitive adaptability in older adults, particularly during repetitive tasks that require inhibitory control. The significant reduction in the response time observed in the PA group suggests that habitual physical activity in daily life could enhance executive adaptability. These findings have practical implications for the design of community-based cognitive health programs and the development of public health interventions aimed at promoting cognitive longevity.

Nevertheless, this study has some limitations. First, the relatively small sample size and cross-sectional design constrain the generalizability of the findings and preclude inferences about temporal or causal relationships. Second, potential confounding factors—such as diet, sleep quality, and psychosocial variables—were not fully controlled and may have influenced both physical activity and cognitive outcomes. Third, this study utilized a single executive function measure, the Stroop task, which does not capture other dimensions, such as working memory or set shifting. Fourth, fNIRS captures only surface-level cortical activation and does not reflect subcortical or network-wide activity. Finally, the physical activity classification was based on self-reported IPAQ data, which may have been subject to recall or reporting bias.

Future research should incorporate more objective assessments of physical activity, such as accelerometers or wearable devices, and utilize neuroimaging modalities, such as fMRI or multimodal approaches (e.g., electroencephalography-fNIRS), to enhance spatial and temporal resolution. Moreover, longitudinal or interventional studies comparing the effects of aerobic, resistance, flexibility, and balance-based exercise programs will further clarify how various exercise modalities influence brain function and cognitive aging trajectories.

The current findings reinforce existing literature demonstrating the cognitive benefits of physical activity in older adults. This study illustrates that lifestyle-based physical activity levels could positively affect adaptive performance in executive function tasks. The observed improvements in the PA group may reflect heightened sensitivity to attentional control and inhibitory processing, contributing to greater cognitive flexibility and strategic response efficiency. These insights provide a valuable foundation for developing scientifically informed and practically feasible intervention strategies to promote cognitive health in the aging population.

## Conclusion

This study investigated the effect of physical activity levels on cognitive performance and prefrontal neurophysiological responses during repeated inhibitory control tasks, specifically the Stroop task, in older adults. The PA group demonstrated significant behavioral improvements in reaction time with repeated task performance, suggesting enhanced learning effects and cognitive adaptability to selective inhibition. In contrast, although no statistically significant group differences were observed in prefrontal HbO changes, a temporal trend was apparent. This discrepancy implies that, although behavioral enhancements were evident, the lack of significant neural differences may be attributable to the technical limitations of fNIRS, such as restricted measurement depth and individual variability in neural recruitment strategies.

Our findings provide experimental evidence that regular and habitual physical activity in older adults is associated with improved behavioral adaptability in executive function tasks and may serve as an effective intervention component for maintaining or enhancing cognitive function. Thus, physical activity may extend beyond physical health maintenance and act as a multifaceted resource that directly or indirectly supports higher-order brain information processing.

Future cognitive health programs targeting older populations should prioritize the integration of diverse exercise modalities and promote sustained, lifestyle-based physical activity. From a policy perspective, proactive measures are warranted to encourage habitual physical activity. Furthermore, longitudinal research employing advanced neurophysiological techniques and objective activity-monitoring tools is needed to elucidate the causal pathways linking physical activity with brain function.

## Data Availability

The datasets presented in this study can be found in online repositories. The names of the repository/repositories and accession number(s) can be found in the article/Supplementary material.
