# Correction: Differential effects of physical activity on behavioral and prefrontal responses during repetitive inhibitory control in older adults

**DOI:** 10.3389/fnagi.2025.1768142

**Published:** 2026-01-12

**Authors:** Jae-Hoon Lee, Minchul Lee, Min-Seong Ha

**Affiliations:** 1 Department of Sport Science, College of Arts and Sports University of Seoul, Seoul, Republic of Korea; 2 Department of Sports Medicine, College of Health Science CHA University, Pocheon-si, Republic of Korea; 3 Laboratory of Sports Conditioning: Nutrition Biochemistry and Neuroscience, Department of Sport Science, College of Arts and Sports University of Seoul, Seoul, Republic of Korea

**Keywords:** physical activity, prefrontal cortex, fNIRS, inhibitory control, older adults

In the published article, there was a mistake in the Funding statement.

The correct Funding statement should read:

The author(s) declared that financial support was received for this work and/or its publication. This study was supported by the Ministry of Education of the Republic of Korea and the National Research Foundation of Korea (NRF), Grant No. NRF-2022S1A5B5A17049589.

The original version of this article has been updated.

