# Supplementary material for: Differential effects of physical activity on behavioral and prefrontal responses during repetitive inhibitory control in older adults
Source: Front Aging Neurosci. 2025 Oct 15;17:1684331. doi: 10.3389/fnagi.2025.1684331 (PMC12571742; doi:10.3389/fnagi.2025.1684331)
Supplement: Supplementary file 1 [file Data_Sheet_1.docx]

| **Supplementary Table 1. Stroop practice task reaction time (PA = 18; PI = 19)** | | | | | | |  |
| --- | --- | --- | --- | --- | --- | --- | --- |
| **Variable** | **Group** | **Practice 1** | **Practice 2** | **Practice 3** | **F-value(p)** | | **η²p** |
| Practice Stroop  RT | PA | 1.34±0.35 | 1.14±0.30*** | 1.10±0.29*** | time | 8.212(.001) | 0.186 |
|  |  |  |  |  | Group | 4.676(.038) | 0.0115 |
|  | PI | 1.24±0.35 | 1.24±0.41 | 1.17±0.33 |  |  |  |
|  |  |  |  |  | time*group | 4.194(.019) | 0.104 |
| Values are presented as means ± standard deviation. ***p < .001 vs practice 1. RT, reaction time; PA, physically active; PI, physically inactive. | | | | | | | |

| **Supplementary Table 2. Changes in Brodmann areas over time (PA = 18; PI = 19)** | | | | | | | | |
| --- | --- | --- | --- | --- | --- | --- | --- | --- |
| **Brodmann area** | **Group** | **Practice 1** | **Practice 2** | **Practice 3** | | **F-value(p)** | | **η²p** |
| **Left DLPFC** | **PA** | **0.0175±0.0567** | **0.0018±0.0677** | | **0.0003±0.0697** | **time** | **3.493(0.037)** | **0.091** |
|  |  |  |  |  |  | **group** | **0.010(0.921)** | **0.0003** |
|  | **PI** | **0.0243±0.0354** | **0.0089±0.0522** | | **0.0009±0.028** |  |  |  |
|  |  |  |  |  |  | **time*group** | **0.161(0.852)** | **0.005** |
| **Right DLFPC** | **PA** | **0.0145±0.0646** | **0.0013±0.0376** | | **0.0163±0.0562** | **time** | **1.467(0.238)** | **0.040** |
|  |  |  |  |  |  | **group** | **2.265(0.142)** | **0.061** |
|  | **PI** | **0.0094±0.0609** | **-0.0132±0.0559** | | **-0.0125±0.0687** |  |  |  |
|  |  |  |  |  |  | **time*group** | **0.189(0.828)** | **0.005** |
| **Left  FPC** | **PA** | **0.0265±0.0412** | **0.0236±0.0391** | | **0.0323±0.0345** | **time** | **0.646(0.528)** | **0.018** |
|  |  |  |  |  |  | **group** | **2.405(0.130)** | **0.064** |
|  | **PI** | **0.0209±0.0584** | **0.0046±0.0589** | | **0.0047±0.0371** |  |  |  |
|  |  |  |  |  |  | **time*group** | **2.125(0.127)** | **0.057** |
| **Right FPC** | **PA** | **0.0225±0.0511** | **0.0142±0.0407** | | **0.0176±0.0416** | **time** | **1.459(0.239)** | **0.040** |
|  |  |  |  |  |  | **group** | **1.288(0.264)** | **0.0360.** |
|  | **PI** | **0.0063±0.0560** | **-0.0046±0.0590** | | **-0.0065±0.0577** |  |  |  |
|  |  |  |  |  |  | **time*group** | **0.133(0.876)** | **0.004** |
| **Left OFC** | **PA** | **0.0482±0.0627** | **0.0580±0.0544** | | **0.0503±0.0421** | **time** | **0.698(0.501)** | **0.020** |
|  |  |  |  |  |  | **group** | **0.682(0.415)** | **0.019** |
|  | **PI** | **0.0461±0.0469** | **0.0301±0.0600** | | **0.0212±0.0526** |  |  |  |
|  |  |  |  |  |  | **time*group** | **1.061(0.352)** | **0.029** |
| **Right OFC** | **PA** | **0.0382±0.0935** | **0.0028±0.0700** | | **0.0191±0.0761** | **time** | **1.521(0.226)** | **0.042** |
|  |  |  |  |  |  | **group** | **0.037(0.849)** | **0.001** |
|  | **PI** | **0.0225±0.0724** | **0.0186±0.0744** | | **0.0104±0.0629** |  |  |  |
|  |  |  |  |  |  | **time*group** | **0.511(0.602)** | **0.014** |
| **Left VLPFC** | **PA** | **-0.0011±0.0788** | **0.0140±0.0458** | | **0.0063±0.0816** | **time** | **1.338(0.272)** | **0.037** |
|  |  |  |  |  |  | **group** | **1.082(0.308)** | **0.030** |
|  | **PI** | **0.0108±0.0651** | **-0.0204±0.0887** | | **-0.0279±0.0700** |  |  |  |
|  |  |  |  |  |  | **time*group** | **0.163(0.850)** | **0.005** |
| **Right VLPFC** | **PA** | **0.0336±0.0534** | **-0.024±0.0873** | | **-0.0048±0.0493** | **time** | **4.548(0.015)** | **0.115** |
|  |  |  |  |  |  | **group** | **0.043(0.837)** | **0.001** |
|  | **PI** | **0.0220±0.0936** | **-0.0146±0.1000** | | **-0.0292±0.1120** |  |  |  |
|  |  |  |  |  |  | **time*group** | **0.860(0.429)** | **0.024** |
| Values are presented as means ± standard deviations. PA, physically active; PI, physically inactive; DLPFC, dorsolateral prefrontal cortex; FPC, frontopolar cortex; OFC, orbitofrontal cortex; VLPFC, ventrolateral prefrontal cortex. | | | | | | | | |
